# Efficacy and Safety of *Bacillus coagulans* IDCC 1201 for Sleep Improvement in Adults with Sleep Disturbance: A Randomized, Double-Blind, Placebo-Controlled Polysomnographic Study

**DOI:** 10.3390/nu18101525

**Published:** 2026-05-11

**Authors:** Hayoung Kim, Jinho Lee, Won Yeong Bang, Han Bin Lee, Haeseong Park, Eun Ju Yun, Duhyeon Kim, Suengmok Cho, Jinkyu Han, Jin Seok Moon

**Affiliations:** 1 Ildong Bioscience, Pyeongtaek-si 17957, Republic of Korea; young@ildong.com (H.K.); ncare0704@ildong.com (J.L.); yeong0417@ildong.com (W.Y.B.); gksqls9131@ildong.com (H.B.L.); haeseong@ildong.com (H.P.); 2 Department of Biotechnology, The Catholic University of Korea, Bucheon 14662, Republic of Korea; ejyun@catholic.ac.kr; 3 Department of Food Science and Technology, Institute of Food Science, Pukyong National University, Busan 48513, Republic of Korea; dueatnow@gmail.com (D.K.); scho@pknu.ac.kr (S.C.); 4 Seoul Special Sleep Center, Seoul 06041, Republic of Korea; sleepmall@nate.com

**Keywords:** *Bacillus coagulans*, probiotics, polysomnography, sleep deprivation

## Abstract

**Background/Objectives**: Sleep continuity is a key determinant of daytime functioning; however, accessible and well-tolerated interventions remain limited. We investigated whether *Bacillus coagulans* IDCC 1201 improves objective sleep continuity and subjective sleep quality in adults with sleep disturbance. **Methods**: In this 4-week, randomized, double-blind, placebo-controlled trial, 80 adults (aged 19–65 years) received *B. coagulans* IDCC 1201 (5.0 × 10^9^ CFU/day) or a placebo; 78 participants completed the study. The primary endpoint was the change in polysomnography-derived sleep efficiency from baseline to week 4. **Results**: Compared with the placebo, *B. coagulans* IDCC 1201 significantly improved sleep efficiency (Δ +13.71 ± 21.14 vs. −0.15 ± 13.35%; *p* = 0.002) and increased sleep duration (total sleep time: Δ +49.56 ± 76.33 vs. −0.50 ± 48.02 min; *p* = 0.002), accompanied by reduced nocturnal wakefulness (wake after sleep onset: Δ −44.40 ± 72.32 vs. +1.88 ± 44.36 min; *p* = 0.003; and total wake time: Δ −12.28 ± 20.22 vs. +0.53 ± 12.32 min; *p* = 0.004). Sleep-stage distribution also favored *B. coagulans* IDCC 1201, with greater increases in stage 2 and REM duration compared with the placebo (between-group *p* = 0.008 and 0.032, respectively). Subjective sleep quality showed greater improvement with *B. coagulans* IDCC 1201 (Pittsburgh Sleep Quality Index change: Δ −3.67 ± 3.37 vs. −1.64 ± 2.83; *p* = 0.036), yielding lower week 4 scores (5.87 ± 2.26 vs. 8.28 ± 3.62; *p* = 0.001). No significant safety concerns were identified. **Conclusions**: These findings indicate that strain-defined probiotic could be used as a nutritional approach for sleep health, particularly for targeting sleep fragmentation and maintenance.

## 1. Introduction

Sleep is an active physiological process regulated by sleep homeostasis and circadian rhythms, and it is essential for recovery and regeneration of physiological and psychological systems including at the neurochemical, hormonal, muscular and immunological levels [1,2]. However, in modern societies, sleep duration has decreased and sleep quality has declined due to factors such as stress, irregular daily rhythms, nighttime use of electronic devices, and the consumption of caffeine and other stimulants [3]. Consistent with these trends, short sleep duration and poor sleep quality among adults are increasingly recognized as global public health concerns, with prevalence rising alongside changes in work and lifestyle environments [4,5].

Insufficient sleep and poor sleep quality are associated with daytime sleepiness and reduced performance and are also linked to increased metabolic and cardiovascular risk and adverse mental health outcomes [6]. Nevertheless, sleep research remains challenging because sleep is defined by both subjective experiences and objective physiological changes in electroencephalographic (EEG) activity. Although many nutritional and functional intervention studies have relied on questionnaire-based measures, such as the Pittsburgh Sleep Quality Index (PSQI), these measures are subjective and susceptible to placebo effects, limiting the precise evaluation of intervention effects. Polysomnography (PSG) is considered the gold-standard method for quantifying sleep continuity and sleep-stage architecture (NREM/REM) and is widely used in human intervention trials to provide objective efficacy endpoints [7,8]. Accordingly, incorporating PSG, which objectively quantifies sleep continuity and architecture, would facilitate more reliable mechanistic insight and better-supported clinical interpretation of intervention effects [9].

In the clinical management of sleep disorders, non-pharmacological approaches, including cognitive behavioral therapy, are commonly used, and hypnotic medications are frequently prescribed [10]. However, pharmacotherapy has been consistently associated with concerns regarding adverse effects, including dependence and tolerance, withdrawal symptoms, cognitive impairment, and daytime drowsiness [11]. Additionally, the burden of long-term use and barriers to prescription access can limit sustained treatment. In this context, there is a need for alternative intervention strategies that balance safety and accessibility. One emerging approach is to target the gut microbiota, which can influence host physiology beyond gastrointestinal function through modulation of barrier integrity, immune and inflammatory signaling, and neuroactive metabolite production [12,13,14]. In particular, the gut microbiota has been proposed to affect central nervous system function and behavior via immune–inflammatory pathways and microbiota-derived metabolites [15,16]. Consistent with this, emerging evidence indicates that the gut–brain axis, through bidirectional interactions between the gut microbiota and the brain, may contribute to the regulation of sleep physiology, highlighting the potential of microbiota-based interventions.

The gut microbiota can produce and modulate a range of metabolites, including melatonin, γ-aminobutyric acid (GABA), serotonin, and short-chain fatty acids (SCFAs), and may influence neurochemical systems involved in sleep–wake regulation by modulating immune and inflammatory responses [17]. Notably, GABA, the principal inhibitory neurotransmitter in the brain, is a key neuroactive molecule influenced by the gut microbiota. By reducing neuronal excitability and promoting relaxation, GABA is implicated in sleep initiation and maintenance [18].

*Bacillus coagulans* is a spore-forming probiotic that produces endospores, distinguishing it from many conventional lactic acid bacteria. This endospore-forming capacity confers relatively high resistance to gastric acid, bile salts, and heat- and desiccation-related stress, supporting enhanced storage stability and viability [19]. These properties may enhance gastrointestinal delivery after oral intake and increase its potential applicability in product development. *B. coagulans* has been reported to support gut health, including the regulation of gut microbiota, enhancement of intestinal function and bowel habits, and modulation of immune function and inflammatory responses [20]. These inflammatory responses may be associated with reduced sleep quality and increased fatigue. From a gut–brain axis perspective, microbiota-based interventions that modulate the gut environment and inflammatory signaling may represent a promising strategy to support sleep health [8]. Furthermore, certain *B. coagulans* strains have been suggested to produce GABA and may be associated with sleep-related metabolic pathways, supporting the hypothesis that sleep can be modulated via a gut microbiota–metabolite–neural axis [21,22]. Collectively, this background supports the application of spore-forming probiotics for sleep health based on their stability and safety profiles, as well as their reported benefits for gut health and modulation of inflammatory responses.

A previous preclinical study evaluated *B. coagulans* IDCC 1201 (SPORABLE^®^, Ildong Bioscience, Pyeongtaek-si, Republic of Korea) as a sleep-promoting functional ingredient using both a pentobarbital-induced sleep assay and EEG/electromyography (EMG)-based sleep architecture profiling [18]. In mice, *B. coagulans* IDCC 1201 reduced sleep latency (SL) and increased sleep duration, and mechanistic interrogation showed that these effects were abolished by co-administration of flumazenil, supporting the involvement of GABAA receptor modulation at the benzodiazepine site. In a longer-term EEG study, repeated administration produced a sustained increase in NREM sleep and improved sleep continuity without evidence of tolerance- or withdrawal-like effects while preserving delta activity as an index of sleep quality. Together, these preclinical findings provide biological plausibility to test whether *B. coagulans* IDCC 1201 can improve objectively measured sleep continuity in humans. However, clinical evidence supporting the use of probiotics for sleep disturbance remains limited, particularly with respect to objectively measured sleep outcomes [23]. Previous studies of microbial interventions have relied mainly on subjective questionnaire-based assessments, and the reported effects have varied across strains. In addition, although some probiotic strains, including *Bifidobacterium longum* 1714 and heat-treated *Limosilactobacillus fermentum* PS150, have been investigated in sleep-related conditions, objective findings have remained limited or inconsistent [24,25]. Therefore, further well-controlled studies are needed to evaluate strain-defined probiotics using objective sleep assessments such as polysomnography. The present study was designed to address this gap by evaluating *B. coagulans* IDCC 1201 in a randomized, double-blind, placebo-controlled trial using overnight PSG together with subjective sleep questionnaires. This design is a particular strength of the present study compared with previous studies as it enables assessment of both objective sleep continuity and subjective sleep quality. Therefore, we conducted a randomized, double-blind, placebo-controlled clinical trial to assess the effects of *B. coagulans* IDCC 1201 on PSG-derived sleep outcomes and subjective sleep quality in adults with sleep disturbance.

## 2. Materials and Methods

### 2.1. Study Participant Selection and Recruitment

In this study, 80 healthy participants with sleep disturbances were recruited through voluntary response to a recruitment advertisement until the target sample size was achieved. Individuals who intended to participate visited the P&K Skin Research Center (Seoul, Republic of Korea), received a full explanation of the study, provided written informed consent, and were screened according to the participant selection criteria. The inclusion criteria were as follows: adults aged 19–65 years, a PSQI score ≥ 5, and voluntary signing of a written informed consent form. The exclusion criteria were as follows: (1) severe insomnia (Insomnia Severity Index [ISI] ≥ 22) or minimal insomnia symptoms (Insomnia Severity Index ≤ 7); this eligibility window was applied to include participants with clinically relevant but non-severe sleep disturbance, avoid floor effects in those with minimal symptoms, and exclude individuals with severe insomnia who might require clinical management; (2) clinician-diagnosed sleep-disorder-related conditions (including major psychiatric disorders), such as obstructive sleep apnea requiring treatment, restless legs syndrome, periodic limb movement disorder, depression, narcolepsy, or other clinically significant sleep disorders; (3) major life changes likely to induce excessive stress within 2 weeks before the first visit; (4) sleep-affecting medications or ongoing hormone therapy; and (5) lifestyle- and schedule-related factors that could affect sleep outcomes, including excessive alcohol intake, heavy smoking, high caffeine intake, night-shift work or irregular sleep schedules, and recent transmeridian travel. Screening for sleep disorder exclusions was based on medical history and questionnaires; respiratory indices obtained from the study PSG were analyzed as exploratory outcomes.

Participants were randomly classified into the *B. coagulans* IDCC 1201 or placebo group at the first visit. The study was approved by the Institutional Review Board (IRB) of the P&K Skin Research Center (IRB No. P2501-7826), in accordance with the Declaration of Helsinki. This study was registered with the Clinical Research Information Service of the Republic of Korea (CRIS No. KCT0011568).

### 2.2. Study Design

**Figure 1:**
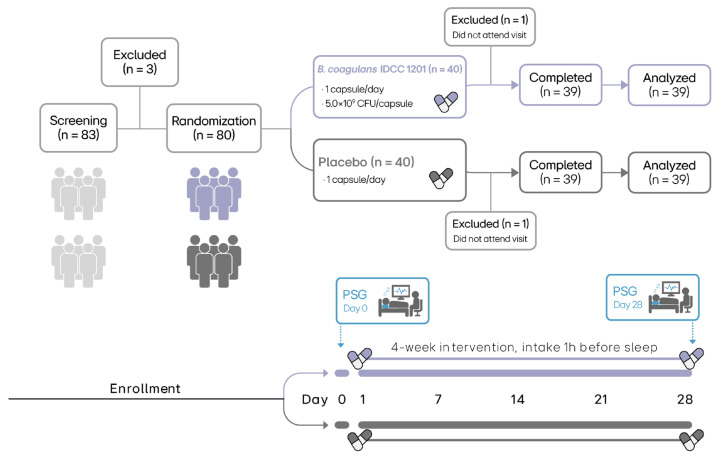
Study flow diagram and trial schedule. A total of 83 participants were screened and 80 were randomized to receive either *Bacillus coagulans* IDCC 1201 (5.0 × 10^9^ CFU per capsule) (*n* = 40) or placebo (*n* = 40). Two participants (one in each group) did not attend a study visit and were excluded, resulting in 39 participants per group that completed the intervention and were included in the final analysis. Allocation was performed using 1:1 block randomization, and study products were dispensed according to subject-specific randomized numbers. PSG, polysomnography.

This study was a randomized, double-blind, placebo-controlled clinical trial (Figure 1). Participants were allocated in a 1:1 ratio to the placebo or *B. coagulans* IDCC 1201 group (*n* = 40 per group) using block randomization. To minimize predictability of treatment assignment, the block size and number of blocks were not disclosed. The randomization list was generated by a monitor or an independent third party not otherwise involved in the conduct of the study. To ensure allocation concealment, subject-specific allocation assignments were prepared according to the randomization list, sealed in opaque envelopes, and supplied to the principal investigator, who retained them in sealed form until study completion. In the event of emergency unblinding, only the allocation for the affected participant could be accessed. The study was conducted in a double-blind manner, and both participants and investigators were blinded to treatment allocation. To maintain blinding, the probiotic and placebo capsules were manufactured to be identical in appearance, including shape, size, and color.

The primary endpoint was the change from baseline (visit 2) to week 4 (visit 3) in PSG-derived sleep efficiency (SE). Key secondary endpoints included changes in sleep latency (SL), total sleep time (TST), wake after sleep onset (WASO), delta power, total PSQI score, Stanford Sleepiness Scale (SSS), Epworth Sleepiness Scale (ESS), Insomnia Severity Index (ISI), Perceived Stress Scale (PSS), Restorative Sleep Questionnaire–Weekly (RSQ-W), and serum GABA concentrations.

Additional PSG indices, including total wake time (TWT), REM latency, sleep-stage duration (NREM stages 1–3, and REM), Respiratory disturbance index (RDI), REM supine RDI, NREM supine RDI, REM lateral RDI, NREM lateral RDI, Obstructive apnea arousal index (Obs AI), Central apnea arousal index (Cent AI), Hypopnea index (HI), Arousal index (Ar.I), Respiratory effort-related arousal (RERA, FI.Ar.I), Spontaneous arousal index (Spon Ar.I), and Total arousal index (Total Ar.I) were assessed as secondary/exploratory outcomes. Efficacy assessments were performed at visit 2 (baseline) and visit 3 (week 4), and outcomes were analyzed as pre-specified.

### 2.3. Treatment Intervention and Compliance

During the 4-week intervention, participants in the *B. coagulans* IDCC 1201 group received 5.0 × 10^9^ CFU/capsule (one capsule/day) of *B. coagulans* IDCC 1201, whereas those in the placebo group received a placebo capsule (one capsule/day) 1 h prior to bedtime. The daily dose of *B. coagulans* IDCC 1201 was determined based on a previous preclinical study demonstrating sleep-promoting effects in mice in which efficacy was observed with 1.0 × 10^8^ CFU of *B. coagulans* IDCC 1201 [18,26,27]. The animal dose was translated to a human equivalent dose (HED) using allometric scaling [26,27]. Based on the HED, the estimated intake was 3.6 × 10^8^ CFU/kg, which was reduced by applying a safety factor of 10 to 3.6 × 10^7^ CFU/kg. For a 60 kg adult, this corresponded to 2.16 × 10^9^ CFU/day. Considering an anticipated effective range of 2–16 times this estimated daily intake, as well as manufacturing yield and recovery of the indicator component, the final study dose was set at 5.0 × 10^9^ CFU/capsule.

Participants were withdrawn if compliance fell below 80% (capsule intake) or if a severe adverse event, as judged by the investigator, occurred. Withdrawal was also permitted at the participant’s request in cases of protocol violations or upon use of medications or supplements that could affect study outcomes. All withdrawals were recorded in the case report forms, and safety assessments were conducted for the withdrawn participants. Safety assessments were performed at visits 2 and 3, and the results were analyzed. Compliance was calculated based on the amount of study product returned (remaining portions) and the dosing diaries completed by participants. For the final efficacy analysis, participants with compliance <80% were excluded from the PP population, whereas all randomized participants were retained in the ITT population.

The investigational product contained *B. coagulans* IDCC 1201 as the main ingredient, with maltodextrin and silicon dioxide as excipients. *B. coagulans* IDCC 1201 was cultured in a commercial medium from Ildong Bioscience (Pyeongtaek-si, Republic of Korea). The placebo capsule contained maltodextrin, silicon dioxide, and sky gardenia yellow colorant. Probiotic and placebo capsules were manufactured to be identical in appearance, including shape, size, and color, to maintain blinding. Probiotic or placebo products were orally administered as one capsule once daily for 4 weeks. The remaining unused capsules were returned and counted to evaluate compliance. Compliance was calculated using the following equation:

Compliance (%) = [(number of capsules dispensed − number of capsules returned)/number of capsules expected to be taken] × 100.

### 2.4. Anthropometric and Dietary Assessment

Demographic and anthropometric characteristics were collected at the study visits. Safety assessments included vital signs (blood pressure, pulse rate, and temperature) and blood tests. General participant characteristics, including alcohol consumption and smoking status, were collected through in-person interviews. Dietary intake was assessed using a 24-h recall dietary questionnaire. Physical activity was assessed using the short-form International Physical Activity Questionnaire (IPAQ-SF, 7-day recall version).

### 2.5. Sleep Questionnaire Scales

Self-administered questionnaires were completed by participants at baseline (visit 2) and post-intervention (visit 3). Sleep-related outcomes were assessed using the PSQI, ISI, and RSQ-W. Daytime sleepiness/drowsiness was assessed using the Stanford Sleepiness Scale (SSS) and the Epworth Sleepiness Scale (ESS), and stress-related outcomes were assessed using the Perceived Stress Scale (PSS). The PSQI is a widely validated instrument for assessing subjective sleep quality [28]. The SSS was included to assess current state sleepiness, whereas the ESS was used to assess habitual daytime sleepiness across daily situations. The RSQ-W was used to assess restorative sleep over the preceding 7 days. All questionnaires were completed at baseline (visit 2) and post-intervention (visit 3).

### 2.6. PSG

Objective sleep outcomes were assessed using overnight PSG with a commercial system (Embla; Flaga, Reykjavik, Iceland). Participants attended the Seoul Special Sleep Center (Seoul, Republic of Korea) at 21:00 for two overnight PSG assessments: at baseline (visit 2, prior to starting the intervention) and after the 4-week intervention (visit 3). For the week 4 PSG, participants consumed the assigned investigational product 1 h before bedtime, consistent with the daily dosing schedule. PSG recordings were performed from approximately 22:00 to 06:00. After electrode and sensor placement, sleep was recorded continuously with simultaneous video monitoring. Sleep stage variables, including REM, Stage 1, Stage 2, and Stage 3, were manually scored by the examiner based on EEG, EOG, and EMG signals. Derived indices, including SE, SL, TST, WASO, TWT, delta power, REM latency, and respiratory/arousal-related indices, were calculated automatically by the system based on the manually scored sleep stages and events. PSG-derived parameters included measures of sleep continuity and architecture, namely, SE, SL, TST, WASO, TWT, delta power, REM latency, and the duration of sleep stages (NREM stages 1–3 and REM). Respiratory- and arousal-related measures were also obtained, including the respiratory disturbance index (RDI; supine and lateral positions in REM and NREM), obstructive and central apnea arousal indices, hypopnea index, arousal index, respiratory effort-related arousals, spontaneous arousal index, and total arousal index.

### 2.7. Serum GABA Measurement

Serum GABA (gamma-aminobutyric acid) concentrations were measured using a Human GABA ELISA kit (BIOMATIK, Kitchener, ON, Canada; EKF57616) according to the manufacturer’s instructions. Because GABA was not analyzed using an automated routine analyzer in this study, an immunoassay-based method was used. The assay quantification range was 3.13–200 ng/mL (lower limit of quantification: 3.13 ng/mL; upper limit of quantification: 200 ng/mL). Serum samples were diluted 1:10 prior to analysis. Samples were analyzed in singlicate. Whole blood was collected for serum preparation, allowed to clot, and centrifuged at 3000 rpm for 10 min at 4 °C. Serum was collected, aliquoted, and stored at −80 °C until analysis. Absorbance was read at 450 nm using a microplate reader (EPOCH2; BioTek, Winooski, VT, USA).

### 2.8. Sample Size Estimation and Statistical Analysis

The sample size required for the study was calculated to be 32 participants per group (two-sided α = 0.05; power = 80%) based on the change in SE. Allowing for attrition, 40 participants were enrolled per group. The analysis populations were defined as follows: the intention-to-treat (ITT) population comprised all randomized participants, regardless of study product intake or protocol adherence. The per-protocol (PP) population comprised participants included in the ITT population who completed the study up to visit 3 without major protocol deviations. Efficacy analyses were primarily performed in the PP population, and ITT analyses were conducted as supportive analyses. For the ITT analysis, missing values were handled using multiple imputations.

Efficacy endpoints were evaluated by comparing baseline (visit 2) and end-of-intervention (visit 3) values. Changes from baseline were calculated for each endpoint. For continuous efficacy endpoints, normality was assessed using the Shapiro–Wilk test. When baseline imbalance was identified, ANCOVA with the baseline value as a covariate was additionally applied. Between-group comparisons were then conducted using an independent *t*-test for normally distributed variables or the Mann–Whitney U test for non-normally distributed variables. Because the change in systolic blood pressure differed significantly between groups, additional ANCOVA models including systolic blood pressure as a covariate were performed as sensitivity/exploratory analyses for efficacy endpoints (rank-based ANCOVA was used when normality assumptions were not met), rather than as the primary analytic model.

Within-group changes were evaluated using paired *t*-tests or Wilcoxon signed-rank tests, as appropriate. Categorical variables were analyzed using the chi-squared or Fisher’s exact test. The association between objective sleep parameters and subjective questionnaire measures was assessed using Pearson’s correlation analysis. Unless otherwise stated, no adjustment for multiple comparisons was applied. Because the study was powered for a single primary endpoint (change in SE), *p*-values for secondary and exploratory endpoints were considered nominal and interpreted descriptively.

## 3. Results

### 3.1. Participants and Compliance

Participant registration began on 3 February 2025, and follow-up and study completion were finalized on 2 July 2025. As shown in Figure 1, 83 participants were evaluated; however, three participants were excluded. Finally, 80 participants (40 in the placebo and 40 in the probiotics group) were enrolled. Consequently, one participant from each group (total: two) dropped out; thus, 78 participants (39 in the placebo and 39 in the probiotics group) completed the clinical trial. Table 1 presents the demographic characteristics of the participants, revealing no significant differences between groups regarding sex, age, height, weight, blood pressure, and heart rate.

Smoking and alcohol consumption can influence sleep [29]. In this study, a statistical analysis of participant characteristics regarding smoking and alcohol consumption was conducted. The results indicated the absence of significant differences between groups. Moreover, the placebo and *B. coagulans* IDCC 1201 groups showed similar compliance rates with study product intake (99.24% and 98.78%, respectively; *p* = 0.829).

### 3.2. PSG Outcomes

**Figure 2:**
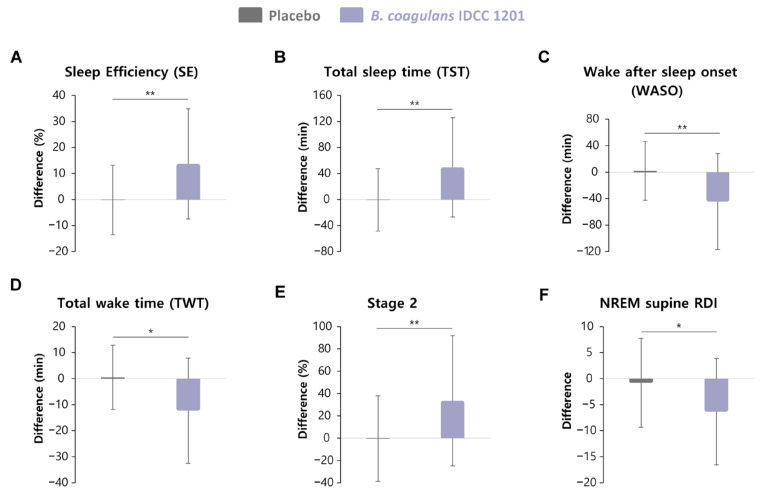
Changes in polysomnography (PSG)-derived sleep outcomes from baseline to week 4 in the placebo (gray) and *Bacillus coagulans* IDCC 1201 group (purple). Box plots show the mean difference between baseline and week 4 for (**A**) sleep efficiency (SE), (**B**) total sleep time (TST), (**C**) wake after sleep onset (WASO), (**D**) total wake time (TWT), (**E**) stage 2 sleep, and (**F**) NREM supine respiratory disturbance index (RDI). Data are expressed as mean ± standard deviation. * *p* < 0.05 and ** *p* < 0.01 vs. placebo (ranked ANCOVA).

PSG-derived sleep outcomes are summarized in Table 2, Figure 2 and Supplementary Table S1.

After 4 weeks, *B. coagulans* IDCC 1201 improved objective sleep continuity relative to the placebo. SE increased by +13.71 ± 21.14% in the *B. coagulans* IDCC 1201 group versus −0.15 ± 13.35% in the placebo group (between-group difference in change: +13.86%; *p* = 0.002). TST increased by +49.56 ± 76.33 min with *B. coagulans* IDCC 1201 compared with −0.50 ± 48.02 min with placebo (between-group difference in change: +50.06 min; *p* = 0.002). Nocturnal wakefulness decreased substantially with *B. coagulans* IDCC 1201, including reductions in WASO (−44.40 ± 72.32 min vs. +1.88 ± 44.36 min; between-group difference in change: −46.28 min; *p* = 0.003) and TWT (−12.28 ± 20.22 min vs. +0.53 ± 12.32 min; between-group difference in change: −12.81 min; *p* = 0.004).

Regarding sleep architecture (Table 2), *B. coagulans* IDCC 1201 increased stage 2 (N2) sleep and REM sleep duration compared with the placebo when changes from baseline were compared between groups (N2: *p* = 0.008; REM: *p* = 0.032). In contrast, delta power and stage 3 (N3) sleep did not show meaningful changes during the intervention period.

Respiratory- and arousal-related PSG measures were analyzed as secondary/exploratory outcomes (Table 2 and Supplementary Table S1). Although the between-group difference in overall RDI was not significant, NREM supine RDI decreased more from baseline in the *B. coagulans* IDCC 1201 group than in the placebo group (*p* = 0.007); however, the week 4 values were comparable between the groups.

### 3.3. GABA Concentrations

Circulating (blood) GABA concentrations are summarized in Table 3. Baseline GABA levels did not differ between the groups (placebo: 2.51 ± 0.41 vs. *B. coagulans* IDCC 1201: 2.47 ± 0.37; between-group *p* = 0.628). After 4 weeks, GABA concentrations remained comparable between the groups (2.37 ± 0.48 vs. 2.30 ± 0.44; *p* = 0.469). Within-group analysis showed a significant reduction in GABA from baseline to week 4 in the *B. coagulans* IDCC 1201 group (change: −0.17 ± 0.42; within-group *p* = 0.014), whereas the placebo group exhibited a non-significant trend toward reduction (change: −0.14 ± 0.44; within-group *p* = 0.056). However, the magnitude of the change did not differ between the groups (between-group *p* = 0.739).

### 3.4. Self-Reported Sleep Outcomes

The PSQI improved in both groups over the 4 weeks; however, the improvement was greater in the *B. coagulans* IDCC 1201 group (Table 4). At week 4, the *B. coagulans* IDCC 1201 group had a lower PSQI total score than the placebo group (5.87 ± 2.26 vs. 8.28 ± 3.62; *p* = 0.001), with a larger reduction from baseline (−3.67 ± 3.37 vs. −1.64 ± 2.83; *p* = 0.036). Other questionnaire outcomes (SSS, ESS, ISI, and PSS) improved within the groups, but between-group differences at week 4 or changes from baseline were not statistically significant. The RSQ-W did not change meaningfully and did not differ between groups.

**Figure 3:**
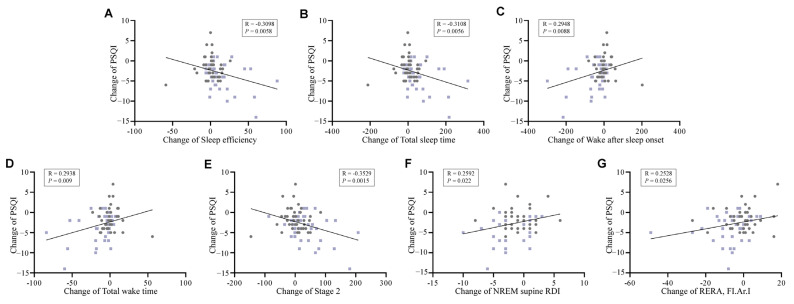
Correlations between changes in PSQI scores and changes in objective sleep parameters. Each panel shows the correlation between change in PSQI score and change in one of the following parameters: (**A**) sleep efficiency (SE), (**B**) total sleep time (TST), (**C**) wake after sleep onset (WASO), (**D**) total wake time (TWT), (**E**) Stage 2, (**F**) NREM supine RDI, and (**G**) RERA, FI.Ar.I. Gray dots indicate the placebo group, whereas purple dots indicate the *Bacillus coagulans* IDCC 1201 group. Statistical significance was assessed using Pearson’s correlation analysis, and *p* < 0.05 was considered statistically significant.

Figure 3 shows the objective sleep parameters that were significantly correlated with changes in PSQI scores. Changes in PSQI scores were significantly negatively correlated with changes in SE, TST, and Stage 2, with *p*-values of 0.0058, 0.0056, and 0.0015, respectively. In contrast, changes in PSQI scores were significantly positively correlated with changes in WASO, TWT, NREM supine RDI, and RERA, FI.Ar.I, with *p*-values of 0.0088, 0.0090, 0.0220, and 0.0256, respectively.

### 3.5. Safety Evaluation

No clinically meaningful safety concerns were identified during the 4-week intervention. No significant adverse events or intervention-related side effects were reported. Clinical laboratory parameters remained stable and did not differ meaningfully between the groups (Supplementary Table S2). Vital signs were generally unchanged from baseline to week 4 in both groups (Table 5).

Although the change in systolic blood pressure showed a statistically significant difference between groups, the values remained within the normal clinical range and were not considered clinically relevant.

## 4. Discussion

In this randomized, double-blind, placebo-controlled study using overnight PSG, 4-week supplementation with *B. coagulans* IDCC 1201 (SPORABLE^®^) was associated with clear improvements in objective sleep continuity. Compared to placebo, the *B. coagulans* IDCC 1201 group showed higher SE and TST, alongside lower WASO and TWT. Because these outcomes were verified using PSG, the findings provide objective support for a sleep continuity benefit of this spore-forming probiotic. The overall pattern is also directionally consistent with preclinical EEG/EMG evidence for *B. coagulans* IDCC 1201 in which repeated administration reduced wakefulness and enhanced NREM sleep continuity over time [18]. Although direct comparisons across species are inherently limited, the convergence of continuity-related outcomes supports the translational relevance of *B. coagulans* IDCC 1201 as a nutritional approach that primarily targets sleep continuity rather than acute pharmacological sedation.

The PSG signature observed here is most compatible with an effect on sleep maintenance and consolidation rather than initiation-only, hypnotic-like effects [30]. Reductions in WASO and TWT indicate fewer and/or shorter awakenings and less fragmentation, which can translate into higher SE and longer TST [29,31]. In parallel, increases in stage 2 (N2) and REM sleep suggest that once nocturnal wake time is reduced, participants may progress more readily into stable sleep states [32]. From a physiological perspective, N2 constitutes a substantial portion of typical adult sleep and often functions as a “buffer” stage that expands when wake time decreases; accordingly, increased N2 coupled with reduced WASO is consistent with improved continuity rather than nonspecific sedation [33]. Similar architecture-level shifts have been described in PSG-based nutraceutical studies where improved continuity is accompanied by N2 expansion without consistent increases in deep sleep. For instance, a placebo-controlled PSG trial of a rice bran extract supplement reported improved SE and TST, followed by an increased N2 percentage, whereas other sleep architecture variables showed limited or inconsistent sensitivity [34]. In contrast, delta power (EEG slow-wave activity) and stage 3 (N3) did not change in either group in the present study. Delta power reflects sleep homeostatic intensity and microstructural quality, which may be less responsive than continuity measures in short interventions or in participants without pronounced slow-wave deficits. Importantly, sleep continuity can improve meaningfully even without increased delta power, particularly if *B. coagulans* IDCC 1201 acts by reducing arousal intrusions rather than by increasing homeostatic sleep pressure.

In the present study, changes in PSQI scores were significantly associated with several objective PSG-derived parameters, suggesting some alignment between perceived sleep improvement and objective changes in sleep continuity and nocturnal arousal-related indices. However, such associations were not evident for all objective sleep variables, and other subjective questionnaire outcomes showed limited significance and little clear correspondence with objective indices. Overall, these results indicate that subjective–objective agreement was selective rather than comprehensive.

Placebo effects and subjective–objective discrepancies are central considerations in sleep intervention research. A meta-analysis focusing on placebo conditions in PSG-based insomnia pharmacotherapy trials reported small-to-moderate placebo-associated improvements, not only in subjective sleep quality, but also in objective PSG variables [35]. Accordingly, questionnaire scores may improve in the placebo arm, especially when participants know that they are being monitored and expect benefits. Furthermore, subjective–objective mismatches are well documented, with frequent discordance between questionnaires and PSG/actigraphy-derived metrics [36,37]. In this context, the robust between-group PSG improvements observed here strengthen the interpretation that *B. coagulans* IDCC 1201 may exert effects beyond expectancy and nonspecific trial participation.

Sleep-related dietary supplements encompass a broad range of functional ingredients, including botanical/food-derived extracts, probiotic or other microbial ingredients, and fermented products, many of which have been investigated for their sleep benefits [38,39]. In particular, multiple extract-based sleep functional ingredients have been developed and clinically evaluated for sleep disturbance [40]. Compared with extract-based approaches, *B. coagulans* IDCC 1201 may offer practical and scientific advantages. First, botanical extracts can show batch-to-batch variability owing to differences in raw materials, harvest conditions, and extraction processes, and the active constituents responsible for sleep effects are not always clearly defined [41,42]. In contrast, *B. coagulans* IDCC 1201 is a strain-defined microbial ingredient, enabling tighter standardization and reproducibility. Second, as a spore-forming *Bacillus*, *B. coagulans* IDCC 1201 is intrinsically stable under typical manufacturing and storage conditions, supporting consistent delivery [43]. Third, many extract-based clinical trials report benefits primarily on subjective sleep quality or sleep initiation [44], whereas the present PSG findings suggest a distinct efficacy profile for *B. coagulans* IDCC 1201—namely, improved sleep maintenance and consolidation. Taken together, these results support *B. coagulans* IDCC 1201 as a reproducible sleep-related functional ingredient with objectively verified improvements in sleep continuity.

In microbe-based sleep functional trials (including probiotics, paraprobiotics, and postbiotics), objective improvements have not been consistently demonstrated across strains and study designs, likely reflecting variability in strain properties, study populations, and endpoint selection. A systematic review in *Clocks & Sleep* noted that although probiotics may improve subjective outcomes (e.g., PSQI) in some studies, objective sleep measures are less consistently assessed and the results remain heterogeneous, limiting firm conclusions regarding physiological effects [45]. This limitation is also illustrated by placebo-controlled trials reporting subjective improvements without corresponding objective changes. For example, *Bifidobacterium longum* 1714 improved aspects of the PSQI and well-being in adults with impaired sleep quality; however, the authors emphasized the absence of positive effects on actigraphy-derived objective metrics [26]. Likewise, a trial of a heat-treated strain (*Limosilactobacillus fermentum* PS150) reported limited overall effects with signals confined to specific subgroups, highlighting the challenge of detecting objective sleep changes in generally healthy participants [24]. Against this background, the present trial provides physiologically grounded evidence for a strain-defined microbial ingredient, demonstrating significant PSG-based improvements in SE, TST, WASO, and TWT. These results reinforce the importance of strain specificity and endpoint selection in microbe-based sleep research and suggest that overnight PSG may be particularly informative for detecting changes in sleep continuity and architecture that may not be fully captured by questionnaires or actigraphy in short-duration studies.

A mechanistic rationale for microbe-based sleep benefits is supported by growing evidence that the gut microbiota can influence sleep through microbial metabolites and neuroactive compounds, including SCFAs, tryptophan–serotonin–melatonin pathways, and GABA-related signaling [46]. Preclinical studies of *B. coagulans* IDCC 1201 reported sleep-promoting effects in mice, including reduced SL and increased NREM sleep, and mechanistic findings implicated GABAergic signaling in the sleep-promoting phenotype [18]. However, in the present trial, blood GABA concentrations did not differ significantly between groups after 4 weeks. This null biomarker result should be interpreted cautiously and does not necessarily negate neurobiological relevance. Peripheral GABA may not adequately reflect central GABAergic tone because sleep-related effects may occur centrally or locally within the gut–brain axis without measurable changes in systemic concentrations. In addition, microbiota-related modulation may influence sleep through vagal afferent signaling, immune–inflammatory pathways, and endocrine mediators rather than through sustained increases in circulating GABA alone [47,48]. Therefore, the absence of a detectable between-group difference in blood GABA does not necessarily exclude biologically meaningful gut–brain signaling relevant to sleep regulation.

Several factors may explain the absence of a detectable between-group difference in blood GABA levels. First, sleep-relevant changes may occur centrally or locally within the gut–brain axis without a corresponding increase in peripheral GABA [47,48,49]. Microbiota-related modulation can influence vagal afferent activity, immune–inflammatory signaling, and endocrine mediators, any of which could affect sleep continuity and architecture independently of circulating GABA. Second, blood sampling at discrete clinic visits (baseline and week 4) may not capture transient or time-dependent changes (e.g., post-dose kinetics or nocturnal fluctuations) that could be more closely aligned with the PSG at night [50,51]. If GABA-related effects are short-lived or circadian-phase dependent, a single pre-post-intervention measurement may underestimate biologically meaningful dynamics. This possibility is indirectly supported by previous findings that direct GABA administration induced measurable physiological responses within 30–60 min after intake, suggesting that GABA-related effects can be time-sensitive [52]. However, because the present study collected blood samples under fasting conditions and evaluated a GABA-producing bacterium rather than GABA itself, such evidence should be interpreted cautiously. In this context, any sleep-related effects of *B. coagulans* IDCC 1201 may have been mediated through gut–brain signaling or local microbial metabolism without requiring a sustained increase in circulating GABA. Third, circulating neurotransmitter measurements are sensitive to pre-analytical and analytical variability (e.g., fasting status, sampling time, processing delays, hemolysis, and matrix effects), and this trial was powered for PSG outcomes rather than biomarker endpoints; thus, modest effects on blood GABA may have been difficult to detect [53,54]. Future studies should incorporate standardized sampling with repeated time points (including pre-dose/post-dose and/or nocturnal sampling), more sensitive quantification methods, and complementary mechanistic readouts (e.g., targeted metabolomics including SCFAs and tryptophan metabolites, inflammatory and stress-related markers, stool neuroactive metabolites, and—where feasible—brain GABA estimates by magnetic resonance spectroscopy). Collectively, the PSG-derived improvements in sleep continuity and PSQI observed here may reflect gut–brain modulation through pathways that are not adequately indexed by circulating GABA alone.

This study also has limitations. The intervention duration was relatively short, and longer trials are needed to evaluate durability and whether slow-wave microstructure (delta power) changes emerge over time. The study did not include microbiome profiling (e.g., metagenomic analysis) or metabolomic biomarkers, which would strengthen the mechanistic interpretation. PSG was recorded on a single night at each time point; therefore, night-to-night variability and the first-night effect could have influenced the estimated sleep parameters, particularly baseline sleep efficiency and other continuity-related indices. Although the same assessment procedure was applied to both groups, these factors may have introduced measurement variability and should be considered when interpreting the magnitude of change. Future studies should incorporate an adaptation night and/or multi-night PSG assessments to improve the reliability of objective sleep measurements. In addition, the study population was predominantly female, representing an important limitation of the present study. Therefore, the findings may not be fully generalizable to male participants or to more sex-balanced populations. Finally, replication in broader non-clinical populations with sleep disturbance, with stratification by baseline fragmentation or hyperarousal, may clarify which subgroups will derive the greatest benefit from *B. coagulans* IDCC 1201.

## 5. Conclusions

In conclusion, this randomized, double-blind, placebo-controlled PSG trial suggests that *B. coagulans* IDCC 1201 (SPORABLE^®^) supplementation for 4 weeks can improve objective sleep continuity in adults with sleep disturbance. Compared with placebo, *B. coagulans* IDCC 1201 was associated with higher SE and longer TST, accompanied by reduced nocturnal wakefulness (including WASO and TWT), indicating improved sleep maintenance and consolidation. Changes in sleep-stage measures were consistent with this continuity-dominant profile, with increases in N2 and REM duration, while slow-wave-related measures (delta power/N3) were not meaningfully altered over the short intervention period. Subjective sleep quality, assessed by PSQI, also improved to a greater extent with *B. coagulans* IDCC 1201, and no clinically meaningful safety concerns were identified.

Together, these findings support the potential of strain-defined probiotic intervention as a nutritional approach for sleep health, particularly for targeting sleep fragmentation and maintenance. Future studies with longer durations, multi-night objective assessments, and mechanistic profiling (e.g., microbiome and metabolomics) are warranted to confirm durability, clarify responder subgroups, and elucidate the pathways underlying the observed PSG improvements.

## Figures and Tables

**Table 1:** Demographic characteristics of the study subjects.

		Placebo (*n* = 39)	*B. coagulans* IDCC 1201 (*n* = 39)	*p* Value
Sex	Male	3 (7.7%)	5 (12.8%)	0.711 ^(4)^
	Female	36 (92.3%)	34 (87.2%)	
Age (years)		46.28 ± 11.80	46.13 ± 10.52	0.877 ^(2)^
Height (cm)		159.22 ± 7.65	160.69 ± 7.37	0.472 ^(2)^
Weight (kg)		61.20 ± 10.70	61.00 ± 9.83	0.988 ^(2)^
Diastolic blood pressure (mmHg)		79.08 ± 8.30	80.31 ± 11.14	0.582 ^(1)^
Systolic blood pressure (mmHg)		112.15 ± 14.84	112.38 ± 15.34	0.946 ^(1)^
Heart rate (bpm)		76.36 ± 9.84	76.28 ± 11.10	0.974 ^(1)^
Smoking	Non-smoker	37 (94.9%)	33 (84.6%)	0.108 ^(4)^
	Ex-smoker	1 (2.6%)	6 (15.4%)	
	Current smoker	1 (2.6%)	0 (0.0%)	
Alcohol drinking	Non-drinker	26 (66.7%)	24 (61.5%)	0.637 ^(3)^
	Moderate drinker	13 (33.3%)	15 (38.5%)	
	Heavy drinker	0 (0.0%)	0 (0.0%)	

Data are expressed as mean ± standard deviation or frequency (proportion). ^(1)^ Independent *t*-test. ^(2)^ Mann–Whitney U test. ^(3)^ Chi square test. ^(4)^ Fisher’s exact test.

**Table 2:** Objective sleep parameters analyzed by PSG.

Parameter		Placebo	*p* Value *	*B. coagulans* IDCC 1201	*p* Value *	*p* Value **	*p* Value **	*p* Value **
Sleep Efficiency, %	At Baseline	85.08 ± 10.21		75.75 ± 21.64		0.049 ^(2)^	0.002 ^†^	0.015 ^(4)^
	At 4 weeks	84.93 ± 14.41		89.46 ± 5.67		0.497 ^(2)^		
	Difference	−0.15 ± 13.35	0.635 ^(3)^	13.71 ± 21.14	<0.001 ^(3)^	0.002 ^(2)^		
Sleep latency, min	At Baseline	14.87 ± 13.41		12.19 ± 11.70		0.401 ^(2)^	0.255 ^†^	
	At 4 weeks	13.50 ± 21.31		7.36 ± 9.28		0.033 ^(2)^		
	Difference	−1.37 ± 22.78	0.128 ^(3)^	−4.83 ± 14.80	0.003 ^(3)^	0.441 ^(2)^		
Total sleep time, min	At Baseline	306.25 ± 36.77		272.88 ± 77.99		0.054 ^(2)^	0.002 ^†^	
	At 4 weeks	305.75 ± 51.84		322.45 ± 20.83		0.401 ^(2)^		
	Difference	−0.50 ± 48.02	0.660 ^(3)^	49.56 ± 76.33	<0.001 ^(3)^	0.002 ^(2)^		
Wake after sleep onset, min	At Baseline	38.87 ± 32.10		75.06 ± 75.50		0.008 ^(2)^	0.009 ^†^	0.130 ^(4)^
	At 4 weeks	40.74 ± 44.43		30.66 ± 17.44		0.901 ^(2)^		
	Difference	1.88 ± 44.36	0.606 ^(3)^	−44.40 ± 72.32	<0.001 ^(3)^	0.003 ^(2)^		
Delta power, %	At Baseline	0.00 ± 0.00		0.00 ± 0.00		1.000 ^(2)^		
	At 4 weeks	0.00 ± 0.00		0.00 ± 0.00		1.000 ^(2)^		
	Difference	0.00 ± 0.00	1.000 ^(3)^	0.00 ± 0.00	1.000 ^(3)^	1.000 ^(2)^		
Total wake time, min	At Baseline	10.79 ± 8.92		20.79 ± 21.11		0.011 ^(2)^	0.011 ^†^	0.130 ^(4)^
	At 4 weeks	11.32 ± 12.34		8.51 ± 4.85		0.885 ^(2)^		
	Difference	0.53 ± 12.32	0.601 ^(3)^	−12.28 ± 20.22	<0.001 ^(3)^	0.004 ^(2)^		
REM latency, min	At Baseline	109.54 ± 60.55		109.59 ± 83.06		0.807 ^(2)^	0.709 ^†^	
	At 4 weeks	89.15 ± 46.88		85.49 ± 48.83		0.379 ^(2)^		
	Difference	−20.38 ± 54.14	0.036 ^(3)^	−23.90 ± 93.57	0.155 ^(3)^	0.783 ^(2)^		
Stage REM, %	At Baseline	55.79 ± 22.96		47.81 ± 27.12		0.516 ^(2)^	0.086 ^†^	
	At 4 weeks	56.81 ± 23.04		66.68 ± 18.37		0.040 ^(1)^		
	Difference	1.01 ± 23.43	0.535 ^(3)^	18.87 ± 30.95	0.001 ^(3)^	0.032 ^(2)^		
Stage 1, %	At Baseline	13.73 ± 6.99		16.81 ± 8.05		0.038 ^(2)^	0.285 ^†^	0.983 ^(4)^
	At 4 weeks	12.67 ± 6.63		13.28 ± 7.20		0.818 ^(2)^		
	Difference	−1.06 ± 7.83	0.276 ^(3)^	−3.53 ± 8.70	0.016 ^(3)^	0.244 ^(2)^		
Stage 2, %	At Baseline	236.72 ± 33.46		207.80 ± 60.20		0.032 ^(2)^	0.007 ^†^	0.111 ^(4)^
	At 4 weeks	236.28 ± 38.64		241.37 ± 24.03		0.901 ^(2)^		
	Difference	−0.44 ± 38.32	0.732 ^(3)^	33.57 ± 58.42	0.001 ^(3)^	0.008 ^(2)^		
Stage 3, %	At Baseline	0.00 ± 0.00		0.46 ± 2.88		0.317 ^(2)^	0.315 ^†^	
	At 4 weeks	0.00 ± 0.00		1.13 ± 7.05		0.317 ^(2)^		
	Difference	0.00 ± 0.00	1.000 ^(3)^	0.67 ± 4.16	0.317 ^(3)^	0.317 ^(2)^		

Data are expressed as mean ± standard deviation. Shapiro–Wilk test was employed to test normality assumption. * *p* values compared within each group. ** *p* values compared between groups. ^(1)^ Independent *t*-test; ^(2)^ Mann–Whitney U test; ^(3)^ Wilcoxon signed rank test; ^(4)^ ranked ANCOVA (covariate: baseline); ^†^ ranked ANCOVA (covariate: change in systolic blood pressure).

**Table 3:** Changes in circulating (blood) GABA concentrations.

Parameter		Placebo	*p* Value *	*B. coagulans* IDCC 1201	*p* Value *	*p* Value **	*p* Value **
GABA	At Baseline	2.51 ± 0.41		2.47 ± 0.37		0.628 ^(1)^	
	At 4 weeks	2.37 ± 0.48		2.30 ± 0.44		0.469 ^(1)^	
	Difference	−0.14 ± 0.44	0.056 ^(2)^	−0.17 ± 0.42	0.014 ^(2)^	0.739 ^(1)^	0.574 ^†^

Data are expressed as mean ± standard deviation. Shapiro–Wilk test was employed to test normality assumption. * *p* values compared within each group. ** *p* values compared between groups. ^(1)^ Independent *t*-test; ^(2)^ paired *t*-test; ^†^ ANCOVA (covariate: change in systolic blood pressure).

**Table 4:** Subjective sleep parameters analyzed by questionnaires.

Parameter		Placebo	*p* Value *	*B. coagulans* IDCC 1201	*p* Value *	*p* Value **	*p* Value **
PSQI	At Baseline	9.92 ± 2.40		9.54 ± 2.65		0.504 ^(1)^	
	At 4 weeks	8.28 ± 3.62		5.87 ± 2.26		0.001 ^(1)^	
	Difference	−1.64 ± 2.83	0.001 ^(3)^	−3.67 ± 3.37	<0.001 ^(3)^	0.036 ^(2)^	0.033 ^‡^
SSS	At Baseline	2.97 ± 1.14		3.05 ± 0.97		0.527 ^(2)^	
	At 4 weeks	2.15 ± 0.81		2.36 ± 1.09		0.366 ^(2)^	
	Difference	−0.82 ± 1.39	0.001 ^(4)^	−0.69 ± 1.13	0.001 ^(4)^	0.735 ^(2)^	0.658 ^‡^
ESS	At Baseline	9.49 ± 4.30		10.31 ± 4.12		0.392 ^(1)^	
	At 4 weeks	6.59 ± 3.86		6.90 ± 4.29		0.644 ^(2)^	
	Difference	−2.90 ± 5.22	0.002 ^(4)^	−3.41 ± 4.63	<0.001 ^(4)^	0.648 ^(1)^	0.872 ^†^
ISI	At Baseline	14.31 ± 4.07		15.13 ± 3.41		0.353 ^(2)^	
	At 4 weeks	8.38 ± 3.72		9.15 ± 4.55		0.313 ^(2)^	
	Difference	−5.92 ± 5.26	<0.001 ^(4)^	−5.97 ± 5.04	<0.001 ^(3)^	0.928 ^(2)^	0.997 ^‡^
PSS	At Baseline	19.10 ± 2.62		18.82 ± 3.46		0.604 ^(2)^	
	At 4 weeks	17.03 ± 3.81		17.28 ± 3.39		0.754 ^(1)^	
	Difference	−2.08 ± 3.59	0.001 ^(3)^	−1.54 ± 3.07	0.004 ^(4)^	0.387 ^(2)^	0.388 ^‡^
RSQ-W	At Baseline	47.65 ± 7.55		47.77 ± 7.43		0.942 ^(1)^	
	At 4 weeks	48.91 ± 8.95		48.15 ± 7.58		0.684 ^(1)^	
	Difference	1.26 ± 10.46	0.455 ^(3)^	0.37 ± 8.60	0.787 ^(3)^	0.683 ^(1)^	0.573 ^†^

Data are expressed as mean ± standard deviation. Shapiro–Wilk test was employed to test normality assumption. * *p* values compared within each group. ** *p* values compared between groups. ^(1)^ Independent *t*-test; ^(2)^ Mann–Whitney U test; ^(3)^ paired *t*-test; ^(4)^ Wilcoxon signed rank test; ^†^ ANCOVA (covariate: change in systolic blood pressure); ^‡^ ranked ANCOVA (covariate: change in systolic blood pressure).

**Table 5:** Vital signs during 4-week intervention period (Safety assessment).

Parameter		Placebo	*p* Value *	*B. coagulans* IDCC 1201	*p* Value *	*p* Value **
Body temperature	At Baseline	36.43 ± 0.27		36.46 ± 0.41		0.671 ^(1)^
	At 4 weeks	36.37 ± 0.26		36.42 ± 0.28		0.427 ^(2)^
	Difference	−0.05 ± 0.34	0.325 ^(3)^	−0.04 ± 0.44	0.567 ^(4)^	0.886 ^(1)^
Diastolic blood pressure	At Baseline	78.79 ± 8.53		79.92 ± 9.81		0.562 ^(2)^
	At 4 weeks	77.38 ± 9.19		79.62 ± 11.07		0.336 ^(1)^
	Difference	−1.41 ± 4.28	0.078 ^(4)^	−0.31 ± 4.65	0.570 ^(4)^	0.279 ^(1)^
Systolic blood pressure	At Baseline	111.31 ± 14.18		110.46 ± 14.89		0.865 ^(2)^
	At 4 weeks	109.79 ± 14.72		112.03 ± 15.84		0.521 ^(1)^
	Difference	−1.51 ± 4.89	0.055 ^(4)^	1.56 ± 6.84	0.162 ^(3)^	0.019 ^(2)^
Pulse	At Baseline	77.51 ± 11.66		78.54 ± 11.85		0.701 ^(1)^
	At 4 weeks	75.90 ± 9.23		76.79 ± 10.94		0.697 ^(1)^
	Difference	−1.62 ± 7.98	0.214 ^(3)^	−1.74 ± 5.83	0.070 ^(3)^	0.429 ^(2)^

Data are expressed as mean ± standard deviation. Shapiro–Wilk test was employed to test normality assumption. * *p* values compared within each group. ** *p* values compared between groups. ^(1)^ Independent *t*-test; ^(2)^ Mann–Whitney U test; ^(3)^ paired *t*-test; ^(4)^ Wilcoxon signed rank test.

## Data Availability

The supporting data for the study can be found in the main manuscript. The supplementary data file contains detailed data on the clinical laboratory examinations associated with the safety of the study. For access to the raw data, please contact the corresponding author with a reasonable request.

## Supplementary Materials

The following supporting information can be downloaded at: https://www.mdpi.com/article/10.3390/nu18101525/s1, Table S1. Respiratory- and arousal-related sleep parameters analyzed by PSG; Table S2. Clinical laboratory safety outcomes during the 4-week intervention period.
