# Supplementary material for: Efficacy and Safety of Bacillus coagulans IDCC 1201 for Sleep Improvement in Adults with Sleep Disturbance: A Randomized, Double-Blind, Placebo-Controlled Polysomnographic Study
Source: Nutrients. 2026 May 11;18(10):1525. doi: 10.3390/nu18101525 (PMC13210199; doi:10.3390/nu18101525)
Supplement: Supplementary file 1 [file nutrients-18-01525-s001.zip › ★Supplementary Table S1.pdf]

Supplementary Table S1. Rsepiratory- and arousal-related sleep parameters analyzed by PSG.

Parameter		Placebo	P value*	*B. coagulans* IDCC 1201	P value*	P value**	P value**
RDI	At Baseline	16.42±7.99		18.20±10.48		0.526^2)	
	At 4 weeks	15.48±7.29		14.72±6.30		0.924^2)	0.183‡
	Difference	-0.93±5.65	0.332^3)	-3.48±6.84	0.004^3)	0.172^2)	
REM supine RDI	At Baseline	30.54±16.23		24.24±21.76		0.068^2)	
	At 4 weeks	26.98±19.94		27.00±14.95		0.646^2)	0.253†
	Difference	-3.56±18.65	0.414^3)	2.76±19.54	0.252^3)	0.148^1)	
NREM supine RDI	At Baseline	16.63±9.83		20.34±13.35		0.194^2)	
	At 4 weeks	15.85±9.22		14.01±8.11		0.487^2)	0.014‡
	Difference	-0.78±8.55	0.660^3)	-6.34±10.23	<0.001^3)	0.007^2)	
REM lateral RDI	At Baseline	14.18±14.78		13.07±17.44		0.374^2)	
	At 4 weeks	15.66±16.58		14.02±12.69		0.840^2)	0.756‡
	Difference	1.47±16.83	0.694^3)	0.95±22.28	0.283^3)	0.621^2)	
NREM lateral RDI	At Baseline	8.59±4.95		10.17±8.28		0.753^2)	
	At 4 weeks	9.10±7.76		9.67±6.96		0.481^2)	0.682†
	Difference	0.51±8.33	0.972^3)	-0.50±5.93	0.392^3)	0.540^1)	
Cent AI	At Baseline	0.29±0.36		0.32±0.57		0.445^2)	
	At 4 weeks	0.31±0.45		0.32±0.39		0.638^2)	0.771‡
	Difference	0.01±0.38	0.935^3)	0.00±0.65	0.706^3)	0.699^2)	
HI	At Baseline	8.68±5.78		10.53±9.85		0.635^2)	
	At 4 weeks	9.37±6.56		8.77±5.83		0.932^2)	0.119‡
	Difference	0.69±4.48	0.455^3)	-1.75±5.91	0.102^3)	0.081^2)	
Ar.I	At Baseline	5.91±4.81		6.85±4.14		0.140^2)	
	At 4 weeks	5.91±4.34		6.07±3.42		0.430^2)	0.543‡
	Difference	-0.01±3.98	0.645^3)	-0.78±3.39	0.306^3)	0.350^2)	
RERA, Fl.Ar.I	At Baseline	5.54±2.94		6.31±3.51		0.292^2)	
	At 4 weeks	4.51±2.02		4.57±2.08		0.818^2)	0.410‡
	Difference	-1.03±2.36	0.005^3)	-1.74±2.84	0.002^3)	0.433^2)	
Spon Ar.I	At Baseline	0.13±0.20		0.08±0.14		0.370^2)	
	At 4 weeks	0.21±0.32		0.09±0.15		0.025^2)	0.441‡
	Difference	0.07±0.41	0.292^3)	0.01±0.19	0.969^3)	0.349^2)	
Total Ar.I	At Baseline	10.09±8.35		9.96±10.30		0.332^2)	
	At 4 weeks	8.79±8.95		8.64±12.39		0.242^2)	0.694‡
	Difference	-1.30±4.25	0.066^3)	-1.32±3.99	0.121^3)	0.838^2)	

Data were expressed as mean ± Standard.
Shapiro-Wilk's test was employed for test of normality assumption.
* P values were compared within each group.
** P values were compared between groups.
1) independent t test
2) Mann Whitney U test
3) Wilcoxon signed rank test
† ANCOVA (covariate : change in systolic blood pressure)
‡ ranked ANCOVA (covariate : change in systolic blood pressure)
