# Supplementary material for: Efficacy and Safety of Bacillus coagulans IDCC 1201 for Sleep Improvement in Adults with Sleep Disturbance: A Randomized, Double-Blind, Placebo-Controlled Polysomnographic Study
Source: Nutrients. 2026 May 11;18(10):1525. doi: 10.3390/nu18101525 (PMC13210199; doi:10.3390/nu18101525)
Supplement: Supplementary file 1 [file nutrients-18-01525-s001.zip › ★Supplementary Table S2.pdf]

Supplementary Table S2. Clinical laboratory safety outcomes during the 4-week intervention period

Parameter		Placebo (n=39)	P value*	*B. coagulans* IDCC 1201 (n=39)	P value*	P value**
	At Baseline	5.87±1.46		5.81±1.46		$0.843^{1)}$
WBC	At 4 weeks	6.00±1.54		5.63±1.12		$0.305^{2)}$
	Difference	0.13±1.57	$0.819^{4)}$	-0.18±1.36	$0.329^{4)}$	$0.351^{1)}$
	At Baseline	4.49±0.34		4.47±0.39		$0.528^{2)}$
RBC	At 4 weeks	4.49±0.31		4.49±0.40		$0.960^{1)}$
	Difference	0.00±0.36	$0.613^{4)}$	0.02±0.28	$0.663^{3)}$	$0.973^{2)}$
	At Baseline	283.43±70.75		280.63±60.71		$0.850^{1)}$
Platelet	At 4 weeks	277.75±68.20		284.18±69.13		$0.836^{2)}$
	Difference	-5.68±47.47	$0.454^{3)}$	3.55±34.39	$0.930^{4)}$	$0.758^{2)}$
	At Baseline	13.35±1.17		13.23±1.44		$0.677^{1)}$
Hemoglobin	At 4 weeks	13.41±1.00		13.24±1.49		$0.569^{1)}$
	Difference	0.05±1.04	$0.752^{3)}$	0.01±0.79	$0.993^{4)}$	$0.711^{2)}$
	At Baseline	40.48±2.82		40.20±3.71		$0.607^{2)}$
Hct	At 4 weeks	40.62±2.66		40.24±3.90		$0.603^{2)}$
	Difference	0.13±2.82	$0.416^{4)}$	0.04±2.59	$0.922^{4)}$	$0.607^{2)}$
	At Baseline	17.15±9.90		17.20±10.40		$0.750^{2)}$
ALT	At 4 weeks	16.68±7.30		17.00±12.92		$0.280^{2)}$
	Difference	-0.48±8.44	$0.651^{4)}$	-0.20±10.44	$0.640^{4)}$	$0.478^{2)}$
	At Baseline	20.40±5.05		21.08±10.34		$0.754^{2)}$
AST	At 4 weeks	20.60±5.14		20.73±8.65		$0.502^{2)}$
	Difference	0.20±4.46	$0.698^{4)}$	-0.35±10.21	$0.809^{4)}$	$0.992^{2)}$
	At Baseline	18.28±10.42		18.73±15.75		$0.467^{2)}$
GGT	At 4 weeks	16.08±8.26		17.53±14.07		$0.962^{2)}$
	Difference	-2.20±6.24	$0.023^{4)}$	-1.20±5.12	$0.029^{4)}$	$0.695^{2)}$
	At Baseline	0.69±0.12		0.70±0.15		$0.761^{2)}$
Creatinine	At 4 weeks	0.68±0.12		0.71±0.14		$0.393^{2)}$
	Difference	-0.02±0.14	$0.518^{4)}$	0.02±0.07	$0.127^{4)}$	$0.167^{2)}$
	At Baseline	98.73±11.58		97.13±8.13		$0.962^{2)}$
Glucose	At 4 weeks	96.40±11.74		95.98±8.12		$0.851^{1)}$
	Difference	-2.33±10.97	$0.161^{4)}$	-1.15±7.16	$0.316^{3)}$	$0.572^{1)}$
	At Baseline	0.56±0.27		0.55±0.26		$0.871^{2)}$
T. Bilirubin	At 4 weeks	0.52±0.29		0.47±0.17		$0.887^{2)}$
	Difference	-0.04±0.23	$0.187^{4)}$	-0.08±0.27	$0.089^{4)}$	$0.830^{2)}$
	At Baseline	90.95±29.49		102.80±63.06		$0.920^{2)}$
CK	At 4 weeks	102.73±64.75		85.75±48.85		$0.028^{2)}$
	Difference	11.78±66.32	$0.906^{4)}$	-17.05±38.63	$0.009^{4)}$	$0.064^{2)}$
	At Baseline	8.14±5.78		7.30±5.04		$0.371^{2)}$
Insulin	At 4 weeks	8.27±5.55		8.91±10.13		$0.627^{2)}$
	Difference	0.13±4.95	$0.791^{4)}$	1.61±10.46	$0.512^{4)}$	$0.430^{2)}$
	At Baseline	125.88±36.37		128.55±39.35		$0.753^{1)}$
LDL-C	At 4 weeks	123.18±34.17		123.63±37.62		$0.955^{1)}$
	Difference	-2.70±24.77	$0.495^{3)}$	-4.93±28.06	$0.274^{3)}$	$0.847^{2)}$
	At Baseline	65.00±16.58		67.40±16.08		$0.513^{1)}$
HDL-C	At 4 weeks	63.10±15.80		68.65±17.16		$0.136^{1)}$
	Difference	-1.90±10.39	$0.254^{3)}$	1.25±10.92	$0.473^{3)}$	$0.190^{1)}$
	At Baseline	203.18±41.61		212.08±44.05		$0.356^{1)}$
T. Cholesterol	At 4 weeks	199.40±41.04		208.93±44.08		$0.600^{2)}$
	Difference	-3.78±23.37	$0.313^{3)}$	-3.15±33.15	$0.181^{4)}$	$0.829^{2)}$
	At Baseline	93.20±38.39		105.33±46.87		$0.268^{2)}$
Triglyceride	At 4 weeks	103.85±48.47		113.38±73.20		$0.784^{2)}$
	Difference	10.65±34.01	$0.061^{4)}$	8.05±55.32	$0.950^{4)}$	$0.178^{2)}$
	At Baseline	140.53±1.80		140.55±1.68		$0.930^{2)}$
Na	At 4 weeks	140.83±1.55		140.85±1.75		$0.844^{2)}$
	Difference	0.30±1.52	$0.214^{4)}$	0.30±1.44	$0.121^{4)}$	$0.775^{2)}$
	At Baseline	4.56±0.42		4.57±0.35		$0.779^{2)}$
K	At 4 weeks	4.57±0.29		4.69±0.45		$0.244^{2)}$
	Difference	0.01±0.37	$0.832^{3)}$	0.12±0.43	$0.084^{4)}$	$0.237^{1)}$
	At Baseline	103.68±1.49		104.20±1.84		$0.177^{2)}$
Cl	At 4 weeks	104.03±1.37		104.25±2.11		$0.635^{2)}$
	Difference	0.35±1.58	$0.145^{4)}$	0.05±1.80	$0.861^{3)}$	$0.430^{1)}$

Data were expressed mean ± standard.
Shapiro-Wilk's test was employed for test normality assumption.
* P values were compared within each group.
** P values were compared between groups.
1) independent *t*-test
2) Mann–Whitney *U* test
3) paired *t*-test
4) Wilcoxon signed rank test
